# Introduction

**DOI:** 10.1186/2193-1801-3-S1-I1

**Published:** 2014-12-04

**Authors:** Tak Yuen, Benjamin Chan

**Affiliations:** Research Support Unit, Headquarters (Academic Services), Vocational Training Council, Kragujevac, Hong Kong

Following the previous two Educational Research Symposiums in 2012 and 2013 which have received overwhelming support from vocational education and training (VET) practitioners within the Vocational Training Council (VTC) in Hong Kong, the scope of this Symposium was expanded and renamed as Practical Social and Industrial Research (PSIR) symposium. The aim of this Symposium was to provide a platform for researchers and practitioners in VET to share and exchange their knowledge and experience with one another. This year’s theme “Advancing VET Practice” incorporated both empirical and practice-based researches that addressed VET issues.

The symposium included 3 keynote speeches entitled “On Translating Technology Research into Industrial Applications” presented by Prof Stephen Wong (Professor, Cornell University), “Assisted Technology for Daily Living” presented by Prof Raymond Tong (Professor, The Chinese University of Hong Kong), “TVET as an Important Factor in Country’s Economic Development” presented by Dr. Margarita Pavlova (Director of UNESCO-UNEVOC Centre Hong Kong, The Hong Kong Institute of Education). After the keynote speech, a total of 3 poster and 9 oral presentations were held covering the sub-themes of “Students and Teachers in VET” and “Teaching and Learning in VET”. Not only the latest research, ideas and innovations that could help to strengthen VET were showcased, evidence-based practices in VET were also shared to inspire practitioners and researchers. The event provided participants with a great opportunity to network with each other and fostered partnership that would support future cooperation.

## Future plans

The PSIR symposium is a regular annual event organised by the Vocational Training Council in Hong Kong. Potential focus areas in 2015 will include vocational education and training, applied research in social innovation or related issues, and environment and sustainability.

